# The prognostic value of the lysyl oxidase family in ovarian cancer

**DOI:** 10.1002/jcla.23538

**Published:** 2020-10-15

**Authors:** Miaomiao Ye, Junhan Zhou, Ying Gao, Shuya Pan, Xueqiong Zhu

**Affiliations:** ^1^ Department of Obstetrics and Gynecology The Second Affiliated Hospital of Wenzhou Medical University Wenzhou China

**Keywords:** Kaplan‐Meier plotter, lysyl oxidase, ovarian cancer, overall survival, prognosis, progression‐free survival

## Abstract

**Background:**

Our study intended to evaluate the prognostic value of lysyl oxidase (LOX) and its four relevant members, the lysyl oxidase–like genes (LOXL1‐4), in ovarian cancer (OC) patients.

**Material and Methods:**

The Kaplan‐Meier plotter (KM plotter) database was used to investigate the prognostic power of the LOX family for OC patients. Overall survival (OS) and progression‐free survival (PFS) were the clinical endpoints. The prognostic roles of the LOX family in OC patients were also analyzed according to various clinicopathological characteristics, including histological subtypes, clinical stages, pathological grades, and chemotherapeutic treatments.

**Results:**

Overexpression of LOX, LOXL1, LOXL2, and LOXL3 mRNA indicated poor OS and PFS in OC patients, particularly in serous and grade II + III OC patients. Overexpression of LOXL4 mRNA resulted in worse PFS in OC patients. Overexpression of LOX and LOXL1 mRNA showed worse OS and PFS in stage III + IV OC patients, and overexpression of LOXL3 mRNA indicated worse OS and PFS in stage I + II OC patients. Overexpression of LOX, LOXL3, and LOXL4 mRNA indicated worse OS and PFS among OC patients who received platinum, taxol, and taxol + platinum chemotherapy. Overexpression of LOXL1 and LOXL2 mRNA was related to lower OS and PFS in OC patients who received platinum chemotherapy.

**Conclusion:**

LOX, LOXL1, LOXL2, and LOXL3 may become potential predictive markers for negative outcomes in OC patients. Moreover, the LOX family can serve as new molecular predictors for the efficiency of platinum‐based chemotherapy in OC patients.

## Graphical abstract

Our study intended to evaluate the prognostic value of lysyl oxidase (LOX) and its four relevant members, the lysyl oxidase‐like genes (LOXL1‐4), in ovarian cancer (OC) patients with various clinicopathological characteristics, which was based on the Kaplan‐Meier plotter database. In conclusion, LOX and LOXL1‐3 are associated with poor prognosis of OC patients. The LOX family predict poor efficiency of platinum‐based chemotherapy in OC patients.

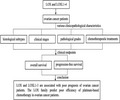

## 1 BACKGROUND

Ovarian cancer (OC), as the fifth cause of cancer‐associated mortality in females in the United States, is considered to be the deadliest malignant carcinoma in gynecology.^1^, ^2^ OC is frequently referred to as a “silent killer” because OC patients remain symptomless until stage III when the disease metastasizes to tissues outside the pelvic cavity.^3^ The gold standard clinical therapy for OC patients is complete debulking surgery followed by chemotherapeutic treatment, which usually involves the combination of paclitaxel‐ and platinum‐based agents.^4^ However, the majority of advanced high‐grade serous ovarian cancer (HGSOC) patients experience disease relapse within 3 years and die within 5 years with the gold standard treatment strategy.^5^ Currently, interval debulking surgery following neoadjuvant chemotherapy is an alternative therapeutic regimen to the gold standard clinical therapy in advanced‐stage OC patients.^6^, ^7^ Therefore, exploring potential prognostic markers for OC patients and probing molecular predictors for the efficiency of chemotherapy regimens in OC patients are necessary.

The lysyl oxidase (LOX) family consists of five members: LOX, the first described member of this family, and its four related members called lysyl oxidase–like genes (LOXL1‐4). The LOX family proteins have two highly conserved sequences in the C‐terminus: a unique copper‐binding (Cu) region and a cytokine receptor–like (CRL) region.^8^ The LOX family proteins are characterized by a variable N‐terminal domain, which was determined to exert distinct functions.^9^ The LOX family is important for extracellular matrix (ECM) cross‐linking and remodeling^10^ and is involved in the process of angiogenesis^11^ and tubulogenesis.^12^ Furthermore, the LOX family has different impacts on the migration,^13^ invasion,^14^ and metastasis^15^ of cancer cells.

Previously published studies have focused on the effects of the LOX family in human tumors and implicated that the LOX family exhibited divergent expression patterns and prognostic functions in diverse cancers.^16^, ^17^, ^18^, ^19^ Low oxygen tension stimulates the activation of the hypoxia‐inducible factor（HIF）pathway in the metastatic microenvironment of ovarian carcinoma and subsequently elevates LOX expression in a HIF‐dependent pattern to facilitate collagen remodeling and tumor invasion.^20^ Wu et al^21^ illustrated that the rs1800449 G473A polymorphism of LOX might increase the susceptibility and recurrence of ovarian carcinoma. Currently, the prognostic functions of the LOX family in patients suffering from OC have not been systematically and comprehensively determined. Therefore, our study intended to evaluate the prognostic power of the LOX family in OC patients, specifically regarding their mRNA expression patterns.

## 2 MATERIALS AND METHODS

This study protocol obtained ethical approval from the ethical committee of the Second Affiliated Hospital of Wenzhou Medical University (No. L‐2020‐08).

### 2.1 Establishment of the ovarian cancer microarray database

The Kaplan‐Meier plotter (KM plotter) database is a freely available online tool (www.kmplot.com) that is capable of assessing the potential impact of cancer‐associated genes on survival for breast cancer,^22^ gastric cancer,^23^ lung cancer,^24^ and ovarian cancer patients.^25^ The available gene mRNA expression data and clinical survival information of 1656 ovarian cancer patients in the KM plotter database were downloaded from the Gene Expression Omnibus (GEO) (http://www.ncbi.nlm.nih.gov/geo/), The Cancer Genome Atlas (TCGA) (http://cancergenome.nih.gov/), and the European Genome‐phenome Archive (EGA) (https://ega.crg.eu/) databases.^26^

### 2.2 Kaplan‑Meier survival analysis

In our study, the prognostic power of the LOX family in patients suffering from OC was assessed by utilizing the KM plotter database. According to higher or lower mRNA expression levels than the automatically selected best cutoff values of the chosen gene, the OC patients were classified into high (upregulation) or low (downregulation) mRNA expression cohorts. Overall survival (OS) and progression‐free survival (PFS) were the studied clinical endpoints, and Kaplan‐Meier survival plots were subsequently generated for the two patient cohorts.^27^ In addition, LOX, LOXL1, LOXL2, LOXL3, and LOXL4 were analyzed for their associations with various clinicopathological features of OC patients, including histological subtypes, clinical stages, pathological grades, and chemotherapeutic treatments.

### 2.3 Statistical analysis

The KM plotter database was developed by using the PostgreSQL server, which can simultaneously process gene expression and clinical data. The data were imported into R software for calculations and analysis. Kaplan‐Meier analysis was performed to draw survival curves, and the log‐rank test was employed to analyze the differences, with *P* < .05 considered statistically significant. The hazard ratios (HRs) with 95% confidence intervals (CIs) were computed. HR > 1 suggested a worse clinical prognosis, and HR < 1 suggested a better clinical prognosis for OC patients. When the 95% CI of the HR contains 1, the results were not considered to be significantly different. Moreover, the KM plotter webpage explained that the generated *P‐*value does not include correction for multiple hypothesis testing by default.^28^

## 3 RESULTS

### 3.1 The available clinical characteristics of OC patients

A total of 1656 OC patients with available gene mRNA expression data and clinical survival information were analyzed in the current study. For LOX, LOXL1, and LOXL2, the OS and PFS curves were based on 1656 and 1435 OC patients, respectively. For LOXL3 and LOXL4, the OS and PFS curves were based on 655 and 614 OC patients, respectively. The available clinical characteristics of OC patients with various histological subtypes, clinical stages, pathological grades, and chemotherapeutic treatments are summarized in Table 1.

**Table 1:** The available clinical characteristics of ovarian cancer patients

Parameters	LOX, LOXL1, LOXL2		LOXL3, LOXL4	
	Cases for OS (n)	Cases for PFS (n)	Cases for OS (n)	Cases for PFS (n)
Histological subtypes				
Serous	1207	1104	523	483
Endometrioid	37	51	30	44
Clinical stages				
Stage I + II	135	163	83	115
Stage III + IV	1220	1081	487	494
Pathological grades				
Grade I	56	37	41	28
Grade II + III	1339	1093	554	476
Chemotherapeutic treatments				
Contains Platin	1409	1259	478	502
Contains Taxol	793	715	357	381
Contains Taxol + Platin	776	698	356	380

Abbreviations: n, number of ovarian cancer patients with available clinical data; OS, overall survival; PFS, progression‐free survival.

### 3.2 Different prognostic values of the LOX family in OC patients

**FIGURE 1:**
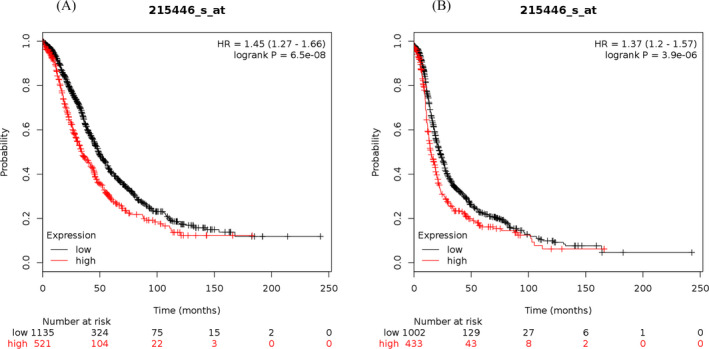
The prognostic value of LOX mRNA expression for the overall survival (OS) and progression‐free survival (PFS) of all ovarian cancer patients. Survival curves were plotted for OS (A, cases = 1656) and PFS (B, cases = 1435)

**FIGURE 2:**
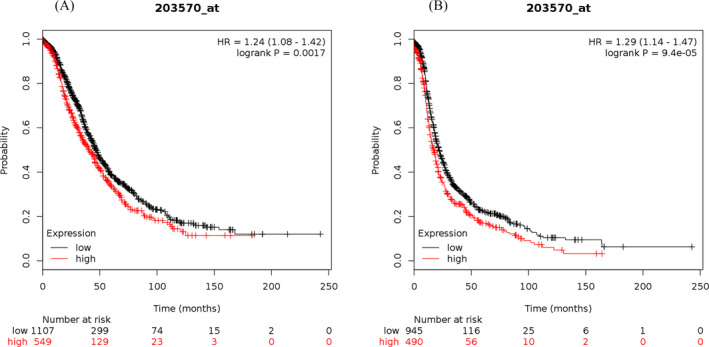
The prognostic value of LOXL1 mRNA expression for the overall survival (OS) and progression‐free survival (PFS) of all ovarian cancer patients. Survival curves were plotted for OS (A, cases = 1656) and PFS (B, cases = 1435)

**FIGURE 3:**
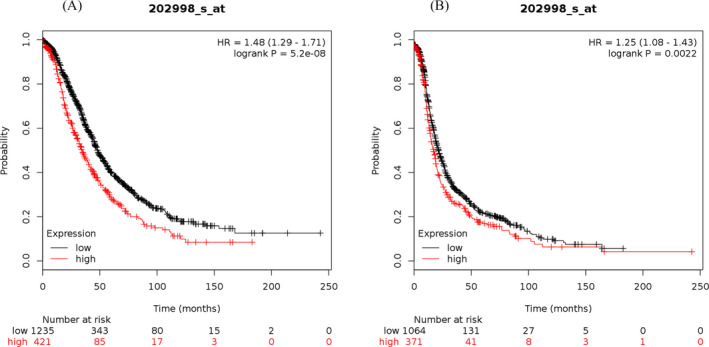
The prognostic value of LOXL2 mRNA expression for the overall survival (OS) and progression‐free survival (PFS) of all ovarian cancer patients. Survival curves were plotted for OS (A, cases = 1656) and PFS (B, cases = 1435)

**FIGURE 4:**
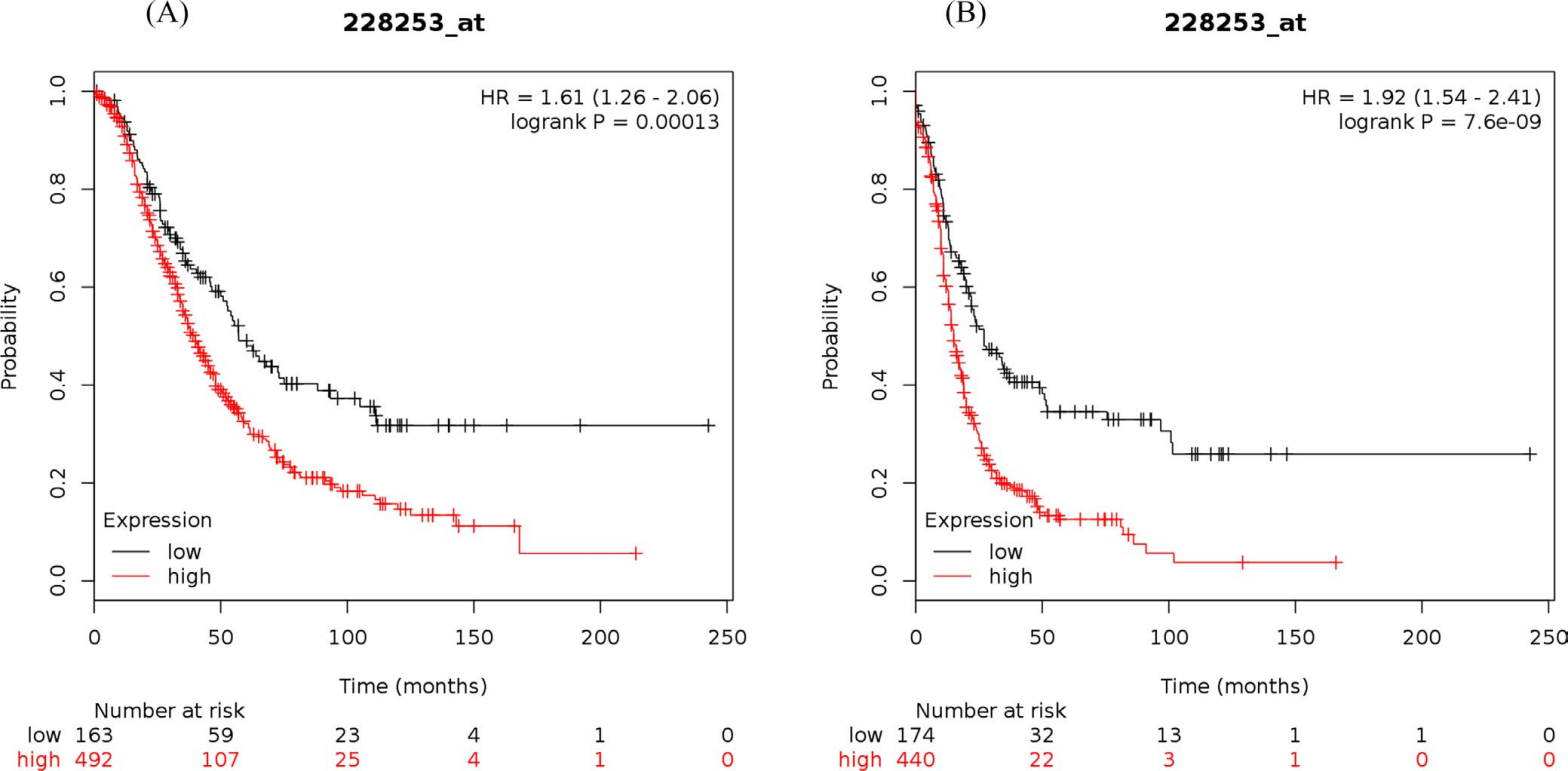
The prognostic value of LOXL3 mRNA expression for the overall survival (OS) and progression‐free survival (PFS) of all ovarian cancer patients. Survival curves were plotted for OS (A, cases = 655) and PFS (B, cases = 614)

**FIGURE 5:**
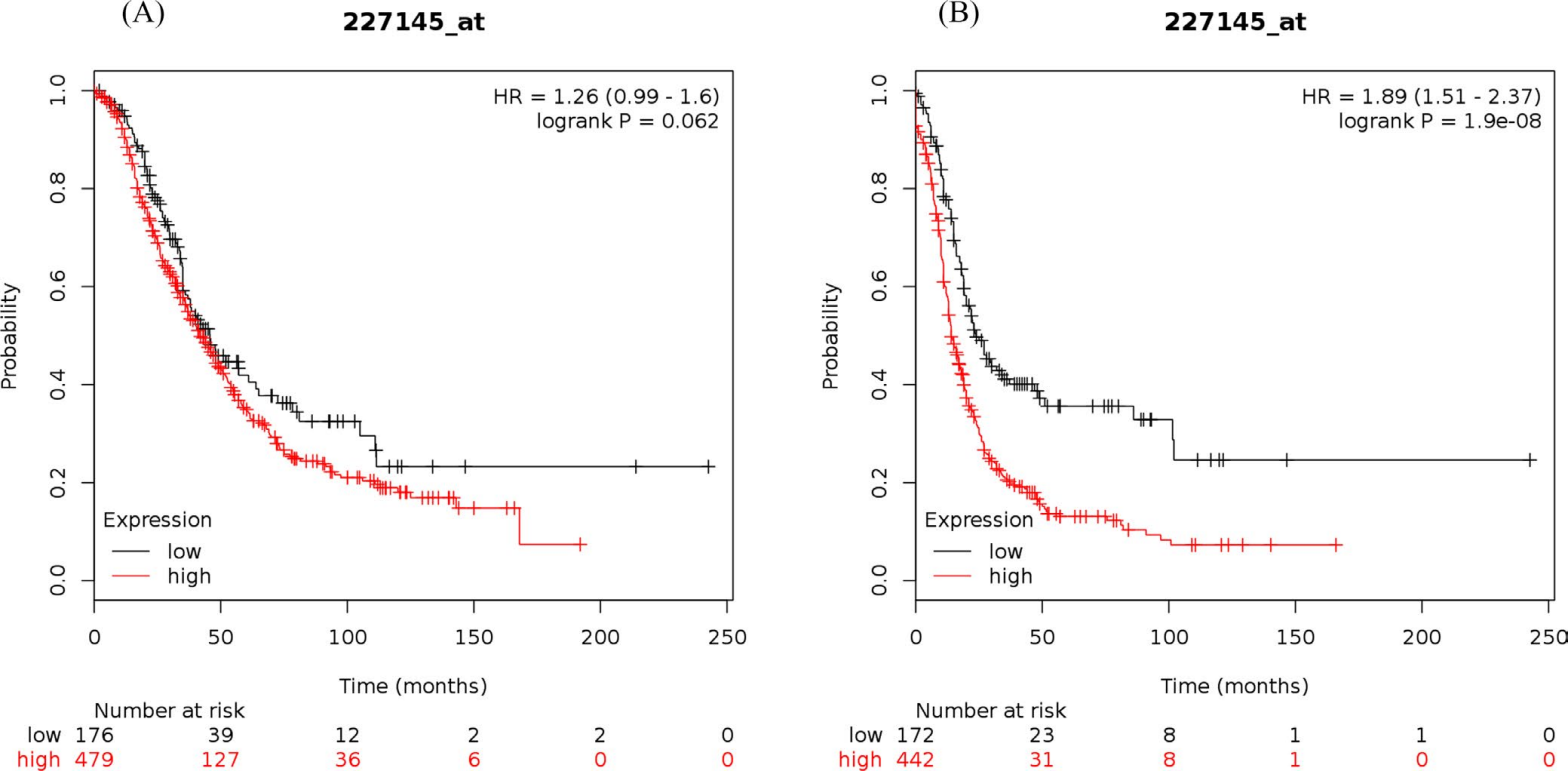
The prognostic value of LOXL4 mRNA expression for the overall survival (OS) and progression‐free survival (PFS) of all ovarian cancer patients. Survival curves were plotted for OS (A, cases = 655) and PFS (B, cases = 614)

The study results showed that the elevated mRNA expression of LOX was associated with lower OS (Figure 1A) and PFS (Figure 1B) in OC patients. The elevated mRNA expression of LOXL1 was demonstrated to be correlated with worse OS (Figure 2A) and PFS (Figure 2B) in patients suffering from OC. The high mRNA expression level of LOXL2 was related to unfavorable OS (Figure 3A) and PFS (Figure 3B) in OC patients. The overexpression of LOXL3 mRNA was correlated with poor OS (Figure 4A) and PFS (Figure 4B) in patients suffering from OC. The correlation of LOXL4 with the OS of OC patients was not significant (Figure 5A), but the elevated mRNA expression of LOXL4 was associated with worse PFS in OC patients (Figure 5B). The median survival times of OC patients with different expression levels of the LOX family members are summarized in Table 2.

**Table 2:** The median survival of ovarian cancer patients with different expression of LOX family

Genes	Expression pattern	Median survival (m)	
		OS	PFS
LOX	Low‐expression cohort	49	22.57
	High‐expression cohort	34.43	14.73
LOXL1	Low‐expression cohort	46.82	22
	High‐expression cohort	40.97	17.38
LOXL2	Low‐expression cohort	48.06	21.43
	High‐expression cohort	34.67	17
LOXL3	Low‐expression cohort	57.1	27
	High‐expression cohort	39.77	15
LOXL4	Low‐expression cohort	45.73	23.82
	High‐expression cohort	41.89	14

Abbreviation: m, months.

### 3.3 Prognostic functions of the LOX family in OC patients with different histological subtypes

The study explored the prognostic functions of the LOX family in serous and endometrioid ovarian carcinoma patients (Table 3). The results indicated that the overexpression of LOX, LOXL1, LOXL2, LOXL3, and LOXL4 mRNA in serous ovarian carcinoma patients was related to unfavorable OS and PFS. When considering endometrioid ovarian carcinoma patients, the mRNA expression levels of LOX, LOXL1, LOXL2, LOXL3, and LOXL4 demonstrated no relation with OS or PFS.

**Table 3:** The prognostic values of LOX family in ovarian cancer patients with different histological subtypes

Genes	Histological subtypes	OS			PFS		
		Cases	HR (95% CI)	*P* ‐value	Cases	HR (95% CI)	*P* ‐value
LOX	Serous	1207	1.55 (1.32‐1.82)	.0000^*^	1104	1.43 (1.23‐1.66)	.0000^*^
	Endometrioid	37	2.89 × 10^8^ (0‐Inf)	.19	51	0.62 (0.24‐1.56)	.3017
LOXL1	Serous	1207	1.2 (1.02‐1.41)	.032^*^	1104	1.29 (1.11‐1.5)	.001^*^
	Endometrioid	37	1.32 × 10^9^ (0‐Inf)	.045	51	2.18 (0.86‐5.53)	.094
LOXL2	Serous	1207	1.39 (1.18‐1.64)	.0000^*^	1104	1.21 (1.03‐1.41)	.023^*^
	Endometrioid	37	8.80 (0.98‐78.78)	.019	51	2.1 (0.83‐5.31)	.11
LOXL3	Serous	523	1.38 (1.06‐1.79)	.016^*^	483	1.49 (1.18‐1.89)	.0000^*^
	Endometrioid	30	1.77 × 10^9^ (0‐Inf)	.037	44	4.26 × 10^8^ (0‐Inf)	.0026
LOXL4	Serous	523	1.32 (1.01‐1.73)	.043^*^	483	1.57 (1.26‐1.96)	.0000^*^
	Endometrioid	30	2.53 (0.26‐24.34)	.41	44	3.13 (0.98‐10.05)	.043

^*^ *P* < .05.

### 3.4 Prognostic power of the LOX family in OC patients with different clinical stages

The study results demonstrated the prognostic correlation of the LOX family with clinical stage I + II (early stages) and clinical stage III + IV (advanced stages) OC patients (Table 4). The overexpression of LOX and LOXL1 mRNA was associated with worse OS and PFS in stage III + IV OC patients. The high expression of LOXL2 mRNA was related to unfavorable OS in stage III + IV OC patients but had no effect on PFS. In addition, the elevated mRNA expression of LOXL3 was associated with lower OS and PFS in stage I + II OC patients and showed worse PFS in stage III + IV OC patients. The upregulated mRNA expression of LOXL4 was illustrated to be associated with worse PFS in stage III + IV OC patients.

**Table 4:** The prognostic values of LOX family in ovarian cancer patients with different clinical stages

Genes	Clinical stages	OS			PFS		
		Cases	HR (95% CI)	*P* ‐value	Cases	HR (95% CI)	*P* ‐value
LOX	I + II	135	1.35 (0.61‐2.98)	.45	163	0.71 (0.39‐1.31)	.28
	III + IV	1220	1.37 (1.17‐1.6)	.0000^*^	1081	1.29 (1.11‐1.5)	.0008^*^
LOXL1	I + II	135	2.17 (0.93‐5.05)	.067	163	0.60 (0.33‐1.1)	.097
	III + IV	1220	1.20 (1.02‐1.4)	.025^*^	1081	1.23 (1.06‐1.43)	.0055^*^
LOXL2	I + II	135	2.04 (0.93‐4.44)	.068	163	0.70 (0.39‐1.26)	.23
	III + IV	1220	1.33 (1.13‐1.57)	.0000^*^	1081	1.16 (0.99‐1.37)	.064
LOXL3	I + II	83	3.27 (1.17‐9.13)	.017^*^	115	3.53 (1.68‐7.38)	.0004^*^
	III + IV	487	1.21 (0.93‐1.57)	.16	494	1.31 (1.04‐1.63)	.019^*^
LOXL4	I + II	83	0.16 (0.02‐1.25)	.047	115	1.62 (0.79‐3.34)	.19
	III + IV	487	0.79 (0.61‐1.03)	.078	494	1.37 (1.11‐1.68)	.0028^*^

^*^ *P* < .05.

The above findings suggested that the elevated mRNA expression of LOX and LOXL1 was associated with worse OS and PFS in stage III + IV OC patients, and the increased mRNA expression of LOXL3 was related to poor OS and PFS in stage I + II OC patients.

### 3.5 Prognostic roles of the LOX family in OC patients with different pathological grades

The study results revealed the prognostic power of the LOX family for pathological grade I (well differentiation) and pathological grade II + III (moderate and poor differentiation) OC patients (Table 5). The elevated mRNA expression of LOX and LOXL1 was demonstrated to be correlated with poor OS and PFS in grade II + III OC patients and showed worse PFS in grade I OC patients. Moreover, the upregulation of LOXL2 and LOXL4 mRNA expression was related to worse OS and PFS in grade II + III OC patients. The overexpression of LOXL3 mRNA was revealed to be associated with unfavorable OS and PFS in OC patients with grade II + III disease and showed worse PFS in OC patients with grade I disease.

**Table 5:** The prognostic values of LOX family in ovarian cancer patients with different pathological grades

Genes	Pathological grade	OS			PFS		
		Cases	HR (95% CI)	*P*‐value	Cases	HR (95% CI)	*P*‐value
LOX	I	56	2.29 (0.87‐6.04)	.084	37	9.66 (2.79‐33.44)	.0000^*^
	II + III	1339	1.38 (1.18‐1.61)	.0000^*^	1093	1.36 (1.17‐1.58)	.0000^*^
LOXL1	I	56	1.68 (0.6‐4.69)	.31	37	4.14 (1.13‐15.1)	.02^*^
	II + III	1339	1.23 (1.05‐1.43)	.0093^*^	1093	1.29 (1.11‐1.5)	.001^*^
LOXL2	I	56	1.76 (0.65‐4.78)	.26	37	3.30 (0.73‐14.91)	.1009
	II + III	1339	1.39 (1.18‐1.63)	.0000^*^	1093	1.23 (1.05‐1.45)	.013^*^
LOXL3	I	41	4.61 (1.01‐20.95)	.03^*^	28	1.52 × 10^9^ (0‐Inf)	.0039
	II + III	554	1.41 (1.1‐1.8)	.0056^*^	476	1.42 (1.15‐1.75)	.0011^*^
LOXL4	I	41	0.73 (0.25‐2.14)	.57	28	0.53 (0.14‐1.96)	.33
	II + III	554	1.34 (1.03‐1.75)	.029^*^	476	1.57 (1.26‐1.94)	.0000^*^

^*^ *P* < .05.

The data above suggested that the increased mRNA expression of LOX, LOXL1, LOXL2, LOXL3, and LOXL4 was related to unfavorable OS and PFS in grade II + III OC patients.

### 3.6 Prognostic significance of the LOX family in OC patients treated with different chemotherapeutic strategies

The study results implicated the prognostic significance of the LOX family in OC patients undergoing platinum‐based chemotherapy, taxol‐based chemotherapy, and taxol + platinum chemotherapy (Table 6). The results elucidated that the high mRNA expression of LOX, LOXL3, and LOXL4 was correlated with a lower OS and PFS in OC patients who were treated with platinum‐based chemotherapy, taxol‐based chemotherapy, and taxol + platinum chemotherapy. Additionally, high LOXL1 and LOXL2 mRNA levels were correlated with unfavorable OS and PFS in OC patients undergoing platinum‐based chemotherapy. The overexpression of LOXL1 mRNA was revealed to be associated with poor PFS, and the overexpression of LOXL2 mRNA was correlated with worse OS in OC patients who were treated with taxol‐based chemotherapy and taxol + platinum chemotherapy.

**Table 6:** The prognostic values of LOX family in ovarian cancer patients with different chemotherapeutic treatments

Genes	Chemotherapeutic treatments	OS			PFS		
		Cases	HR (95% CI)	*P* ‐value	Cases	HR (95% CI)	*P* ‐value
LOX	Contains platin	1409	1.44 (1.24‐1.67)	.0000^*^	1259	1.38 (1.2‐1.59)	.0000^*^
	Contains taxol	793	1.42 (1.16‐1.73)	.0005^*^	715	1.41 (1.19‐1.67)	.0000^*^
	Contains taxol + platin	776	1.39 (1.13‐1.7)	.0015^*^	698	1.42 (1.2‐1.69)	.0000^*^
LOXL1	Contains platin	1409	1.25 (1.08‐1.45)	.0023^*^	1259	1.24 (1.08‐1.41)	.002^*^
	Contains taxol	793	1.19 (0.96‐1.47)	.1	715	1.25 (1.04‐1.49)	.018^*^
	Contains taxol + platin	776	1.18 (0.95‐1.45)	.14	698	1.25 (1.04‐1.5)	.019^*^
LOXL2	Contains platin	1409	1.45 (1.25‐1.7)	.0000^*^	1259	1.17 (1.01‐1.35)	.034^*^
	Contains taxol	793	1.47 (1.2‐1.8)	.0000^*^	715	1.21 (1‐1.47)	.054
	Contains taxol + platin	776	1.46 (1.19‐1.8)	.0003^*^	698	1.16 (0.98‐1.39)	.092
LOXL3	Contains platin	478	1.49 (1.14‐1.96)	.0037^*^	502	1.48 (1.17‐1.87)	.0010^*^
	Contains taxol	357	1.59 (1.12‐2.26)	.0091^*^	381	1.42 (1.08‐1.87)	.011^*^
	Contains taxol + platin	356	1.59 (1.12‐2.27)	.0087^*^	380	1.42 (1.08‐1.86)	.012^*^
LOXL4	Contains platin	478	1.46 (1.09‐1.96)	.012^*^	502	1.65 (1.3‐2.09)	.0000^*^
	Contains taxol	357	1.45 (1.01‐2.09)	.043^*^	381	1.66 (1.26‐2.17)	.0002^*^
	Contains taxol + platin	356	1.46 (1.01‐2.1)	.041^*^	380	1.65 (1.26‐2.17)	.0003^*^

^*^ *P* < .05.

## 4 DISCUSSION

Our research proposed to explore the prognostic functions of the LOX family in OC patients, focusing on the mRNA expression levels. The results showed that the elevated expression of LOXL4 mRNA was related to worse PFS in OC patients, and the elevated expression of LOX, LOXL1, LOXL2, and LOXL3 mRNA was associated with unfavorable OS and PFS in OC patients.

Among this five‐protein family, LOX is the best‐studied isoform. LOX is situated and exerts functions in the nuclei of fibrogenic cells,^29^ catalyzing the covalent cross‐linking of collagens and elastin and subsequently increasing the extracellular matrix tension.^30^ The role of LOX as a potential predictive factor in cancer metastasis and progression was evidenced in several human cancers, such as pancreatic carcinoma,^31^ gastric carcinoma,^32^, ^33^ hepatocellular carcinoma (HCC),^34^ lung adenocarcinoma,^35^ breast cancer,^36^, ^37^ and cervical cancer.^38^ Moreover, the increased expression of LOX facilitated the proliferative, migrative, invasive, and anchorage‐independent growth potential of HGSOC cells.^20^, ^39^, ^40^ Nuclear LOX expression is a detrimental prognostic indicator for advanced HGSOC patients.^39^ To some extent, our results were consistent with the conclusion mentioned above involving ovarian carcinoma. In our study, higher mRNA expression levels of LOX were associated with unfavorable outcomes in OC patients, particularly in serous, grade II + III and stage III + IV OC patients. Overall, LOX may be a predictive indicator of poor outcomes in serous, advanced‐stage, and moderately and poorly differentiated OC patients. Further studies are needed to determine the cellular location of LOXL2 in ovarian cancer cells.

Lysyl oxidase–like 1 (LOXL1) has been elucidated to degrade extracellular pH‐associated matrix, especially in acidic extracellular environments.^41^ To date, only a few studies have clarified the correlation between LOXL1 and cancer development. Wu et al^42^ reported that LOXL1 exerted an antitumor function in human bladder carcinoma by antagonizing the Ras/ERK signaling pathway. However, Lee et al^41^ provided evidence that LOXL1 was overexpressed in metastatic sites compared to primary lung cancer tissues and that the upregulation of LOXL1 promoted lung cancer cell metastasis and invasiveness when extracellular lactate accumulated, suggesting that LOXL1 was an oncogene. Currently, no study has investigated the role of LOXL1 in the outcomes of OC patients. Our research revealed that the upregulation of LOXL1 mRNA expression was related to poor outcomes in OC patients, particularly in serous, grade II + III, and stage III + IV OC patients. Consequently, LOXL1 could be considered a novel prognostic and predictive marker for poor outcomes in OC patients, especially for serous, advanced‐stage, and moderately and poorly differentiated OC patients.

Lysyl oxidase–like 2 (LOXL2) was previously described as a Snail1 regulator and epithelial‐mesenchymal transition (EMT) inducer.^43^ The overexpression of LOXL2 played a tumor‐promoting role and indicated poor prognosis in esophageal squamous cell carcinoma,^16^ hepatocellular carcinoma,^44^, ^45^ lung carcinoma,^17^ gastric carcinoma,^46^ and breast carcinoma.^47^, ^48^ However, no study has explored the function of LOXL2 in ovarian carcinoma. The present research illustrated that the overexpression of LOXL2 mRNA was related to unfavorable outcomes in OC patients, in particular for serous, grade II + III OC patients. The elevated expression of LOXL2 mRNA was demonstrated to be correlated with lower OS in stage III + IV OC patients but revealed no relationship with PFS. Overall, LOXL2 may have a prognostic impact on predicting the negative outcomes of OC patients, but more efforts are needed to further document the correlation of LOXL2 with different clinical stages.

Lysyl oxidase–like 3 (LOXL3) has been discovered in several tissues, such as the chorion^8^ and uterus.^49^ However, studies concerning LOXL3 in cancers are quite limited. The increased expression of LOXL3 was detected in human melanoma and facilitated carcinogenesis.^50^ This research also indicated that LOXL3 was necessary for completing proper mitosis and that the silencing of LOXL3 in melanoma cells triggered cancer cell apoptosis.^50^ Jeong et al^51^ demonstrated that estrogen receptor (ER) and progesterone receptor (PR) expression in breast carcinoma tissues was notably related to the expression of LOXL3, and LOXL3 expression had no relationship with the outcomes of breast carcinoma patients. No investigation has evaluated the function of LOXL3 in ovarian carcinoma. Our study discovered that the upregulated mRNA expression of LOXL3 was related to poor outcomes in OC patients. In particular, the elevated mRNA expression of LOXL3 indicated poor prognosis in serous, grade II + III and stage I + II OC patients. Therefore, higher LOXL3 expression was a predictive indicator of poor clinical prognosis in patients with ovarian carcinoma.

Lysyl oxidase–like 4 (LOXL4) is situated in the cytoplasm and closely connected to the cell membrane.^52^ The role of LOXL4 in human cancers is controversial. A previous study^53^ documented that the downregulated expression of LOXL4 mRNA and protein was detected in HCC tissues, which was demonstrated to be correlated with decreased overall survival and a higher rate of cumulative relapse in HCC patients. However, one study^54^ indicated that LOXL4 was overexpressed in HCC tissues, which suggested poor prognosis in HCC patients. Furthermore, the upregulated expression of LOXL4 was detected in esophageal squamous cell carcinoma^19^ and gastric carcinoma,^55^ which predicted worse survival in these cancer patients. There has been no research studying the role of LOXL4 in ovarian carcinoma. The present investigation revealed that the enhanced mRNA expression of LOXL4 indicated worse PFS in OC patients. Moreover, LOXL4 mRNA overexpression was related to lower OS and PFS in serous and grade II + III ovarian carcinoma patients and poor PFS in endometrioid ovarian carcinoma patients. Nonetheless, the prognostic power of LOXL4 in OC patients with different clinical stages needs further study.

The development of chemotherapeutic resistance has gradually become an essential obstacle to overcome in order to effectively ameliorate the clinical prognosis of cancer patients. Research on the influence of the LOX family on the chemotherapeutic treatment efficiency of OC patients is limited. De Donato et al^39^ stated that elevated nuclear expression of LOX was correlated with platinum‐based chemotherapy resistance in OC patients, which was evidenced by immunohistochemistry staining between platinum‐sensitive and platinum‐resistant OC tissues. One study documented that treatment with the LOXL2‐neutralizing antibody AB0023 in ovarian cancer mice contributed to the normalization of cancer vessels and increased the perfusion of cancer‐related vessels, thus facilitating the delivery of chemotherapeutic drugs into cancers and enhancing the chemotherapeutic efficiency.^56^ Sebban et al^57^ showed that LOXL4 was highly expressed in ovarian cancer following chemotherapeutic treatment with both paclitaxel and platinum compounds. Our current investigation discovered that the elevated expression of LOX family mRNA potentially predicted unfavorable clinical outcomes in OC patients who received platinum‐based chemotherapy. Additionally, increased LOX, LOXL3, and LOXL4 mRNA expression was associated with worse prognosis in OC patients who were treated with taxol‐based chemotherapy or taxol + platinum chemotherapy. Consequently, we proposed that chemotherapeutic drugs combined with LOX family gene‐target inhibitors may represent a new therapy for platinum‐based chemoresistant OC patients, and the combination of LOX, LOXL3, and LOXL4 inhibitors with chemotherapeutic drugs may improve the prognosis of taxol‐based and taxol + platinum chemoresistant OC patients.

In addition, some limitations of our present study also need to be discussed. First, our study documented the prognostic value of the LOX family in OC patients only at the mRNA expression level. We will further study the LOX family at the protein level to certify the prognostic functions of this family in OC patients. Second, the data we collected were merely from a freely available online database, and the mechanisms of how the LOX family exerts its functions on the prognosis of OC patients are unknown. More efforts are needed to further illustrate the mechanisms and pathways of the LOX family that are related to the biological behaviors (metastasis, proliferation, migration, invasion, etc) of ovarian cancer based on biochemical (cellular function), physiological (animal models), and pathological (human cancer specimens) researches.

## 5 CONCLUSION

In summary, LOX, LOXL1, LOXL2, and LOXL3 may be negative prognostic indicators for OC patients, particularly for serous, grade II + III and platinum‐based chemoresistant OC patients. This finding may help to accurately predict the prognosis of OC patients, and the discovery of LOX family gene‐target inhibitors may be an efficient way to adjuvantly treat OC patients.

## CONFLICT OF INTEREST

The authors declare that there are no conflicts of interest.
